# Current RNA strategies in treating cardiovascular diseases

**DOI:** 10.1016/j.ymthe.2024.01.028

**Published:** 2024-01-29

**Authors:** Shirley Pei Shan Chia, Jeremy Kah Sheng Pang, Boon-Seng Soh

**Affiliations:** 1 Institute of Molecular and Cell Biology (IMCB), Agency for Science, Technology and Research (A∗STAR), 61 Biopolis Drive, Proteos, Singapore 138673, Singapore; 2 Department of Biological Sciences, National University of Singapore, 16 Science Drive 4, Singapore 117558, Singapore

**Keywords:** RNA therapeutics, cardiovascular disease, antisense oligonucleotide, RNA interference, CRISPR, genetic variants, therapeutic delivery, lipid nanoparticle, adeno-associated virus

## Abstract

Cardiovascular disease (CVD) continues to impose a significant global health burden, necessitating the exploration of innovative treatment strategies. Ribonucleic acid (RNA)-based therapeutics have emerged as a promising avenue to address the complex molecular mechanisms underlying CVD pathogenesis. We present a comprehensive review of the current state of RNA therapeutics in the context of CVD, focusing on the diverse modalities that bring about transient or permanent modifications by targeting the different stages of the molecular biology central dogma. Considering the immense potential of RNA therapeutics, we have identified common gene targets that could serve as potential interventions for prevalent Mendelian CVD caused by single gene mutations, as well as acquired CVDs developed over time due to various factors. These gene targets offer opportunities to develop RNA-based treatments tailored to specific genetic and molecular pathways, presenting a novel and precise approach to address the complex pathogenesis of both types of cardiovascular conditions. Additionally, we discuss the challenges and opportunities associated with delivery strategies to achieve targeted delivery of RNA therapeutics to the cardiovascular system. This review highlights the immense potential of RNA-based interventions as a novel and precise approach to combat CVD, paving the way for future advancements in cardiovascular therapeutics.

## Graphical abstract

**Figure:**
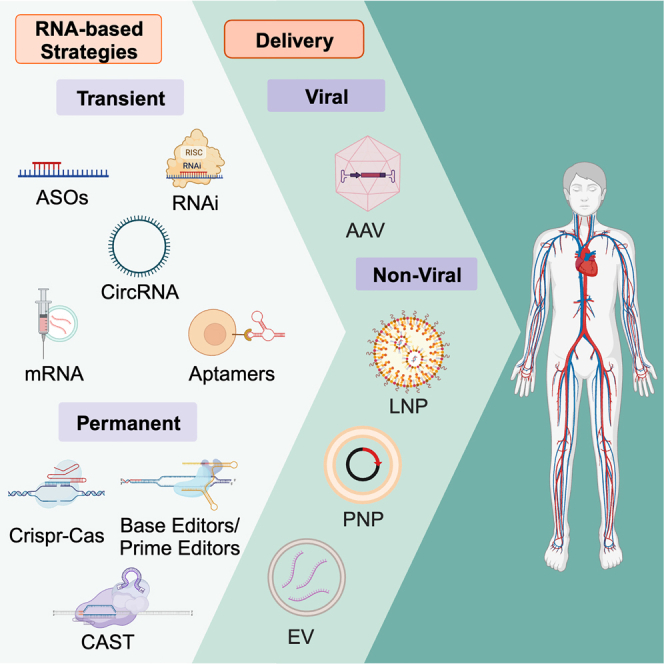

## Teaser

Soh and colleagues explored the promising myriad of RNA-based therapeutics for cardiovascular disease (CVD). Addressing both Mendelian and acquired CVDs, gene targets were identified for precise interventions. The challenges and opportunities of targeted RNA delivery to the cardiovascular system are also highlighted, underscoring RNA’s potential for novel CVD treatments.

## Introduction

Claiming 18 million lives annually, cardiovascular diseases (CVDs) remain the leading cause of mortality worldwide. CVDs encompass a broad spectrum of diseases that affect the heart and vasculature.^1^ Over the decades, significant progress in comprehending the pathogenesis and mechanisms underlying CVD has been made, resulting in remarkable improvements in cardiovascular pharmacology. "First-in-class" cardiovascular drugs, such as mavacamten for hypertrophic cardiomyopathy and SGLT2 inhibitors for heart failure independent of left ventricular (LV) ejection fraction, have obtained approval.^2^ Additionally, numerous randomized clinical trials evaluating the effectiveness and safety of repurposing "old" drugs in combination have been published.^2^ However, we have to recognize that traditional medication offers limited curative effects and primarily focuses on prevention and symptomatic relief.^3^ Oftentimes, prolonged usage of these drugs may also lead to side effects.^3^ As the disease advances, cardiac surgeries like LV assist device implantation or, in extreme cases, heart transplantation—which is scarce—are highly effective but limited by complex procedures and potential postoperative complications.^4^ Failure to treat these diseases leads to heart failure, a condition that impacts 1%–2% of the world’s population, which imposes a substantial societal burden.^5^ As a result, there is a pressing need for a breakthrough in the landscape of CVD treatments and RNA therapeutics could be part of it.

RNA therapeutics is an emerging field of medicine that utilizes RNA-based molecules as drugs to treat various diseases.^6^ Recognized with the recent 2023 Nobel Prize, the incorporation of modified nucleoside bases played a pivotal role in the rapid development of safe and effective messenger ribonucleic acid (mRNA) vaccines for COVID-19. This breakthrough not only propelled the field of mRNA-based prophylactics but also marked a transformative era for RNA therapeutics. Leveraging the adaptability of RNA therapeutics to address evolving disease strains, they offer a unique advantage.^6^ In addition, these therapeutics can be robustly applied to target the different stages of the molecular biology central dogma, modulating expression and activity of proteins of interest.^7^ By leveraging on its versatility, the use of RNA therapeutics could be a major step forward in the treatment of CVD. In this review, we have outlined the RNA strategies available, while proposing potential therapeutic targets in CVD. The different modes and challenges associated with cardiac-specific delivery of RNA-based therapeutics have also been highlighted.

## RNA-based therapeutics

**Figure 1:**
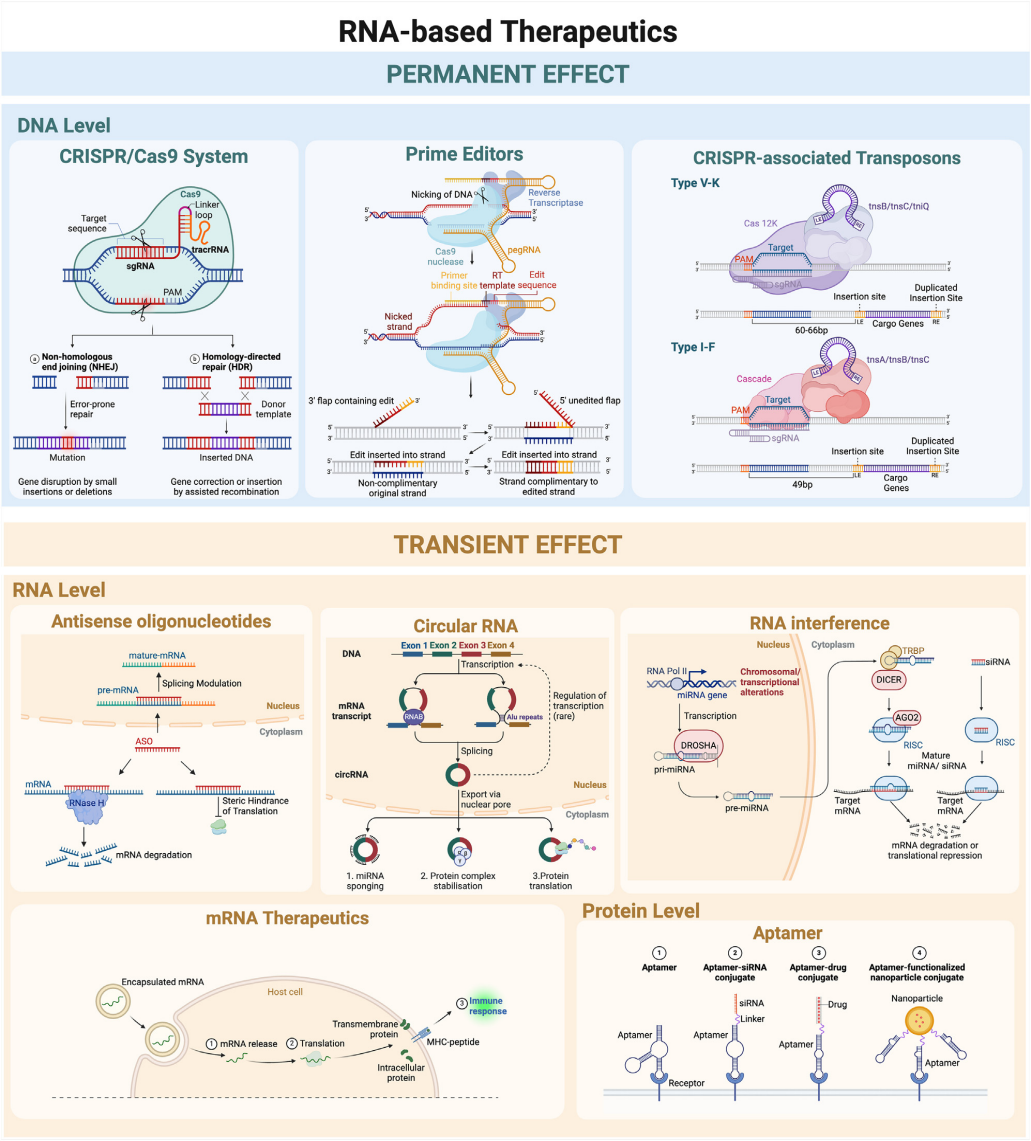
Mechanistic basis of RNA-based therapeutics directed at DNA, RNA, and protein level Created with BioRender.com.

RNA-based therapeutics has an edge over conventional drug strategies, such as small molecules and antibodies, due to their extensive druggable targets across deoxyribonucleic acid (DNA), RNA, and protein levels, coupled with the ease of synthesis, significantly shortening the time "from bench to bedside.” Herein, we have outlined the mechanistic basis of current strategies directed at the different levels, which have been extensively discussed in prior reviews.^8^^,^^9^ Additionally, we introduce potentially novel approaches in this field. A detailed illustration of the mechanism by which these RNA-based therapeutics exert their effects is presented in Figure 1.

### Effectors at the DNA level

As a recent addition to gene-editing tools, clustered regularly interspaced short palindromic repeat (CRISPR)-based systems are classified as RNA-based therapeutics due to their internal RNA components. This CRISPR landscape is dynamically expanding, ushering in a "clinical CRISPR ecosystem" that integrates diverse genome editing methods like base editing, prime editing, and innovative CRISPR proteins (e.g., CRISPR-associated transposons or CASTs) to address a wider range of treatable conditions. Nonetheless, effective delivery of this multi-component system is often hampered by its large size. Presently, this system is delivered in a plasmid expression cassette to cells of interest within a delivery vector, which is discussed in the section of Cardiac-specific delivery of RNA therapeutics.

#### CRISPR-Cas-based genome editing

Originating from prokaryote adaptive immune systems, the CRISPR-associated protein (Cas) systems are readily programmable to target foreign genetic material.^10^ Two categories have been assigned to naturally occurring CRISPR-Cas systems, where class 1 involves multiple effector complexes and class 2 employs a single-protein effector to cleave nucleic acid.^11^^,^^12^ The simpler architecture of class 2 systems made them more preferable for therapeutic genome editing.^13^ Functionally, this system utilizes an engineered guide RNA (gRNA) binding to an RNA-guided Cas nuclease, forming the Cas-gRNA ribonucleoprotein.^14^ This complex then binds to a target site with complementary DNA sequence adjacent to a protospacer-adjacent motif (PAM), essential for preventing unwanted cleavage at matching sites lacking the PAM sequences.^14^ Upon binding of the complex, the double-stranded DNA unwinds and forms an RNA-DNA heteroduplex between the gRNA and the target DNA strand known as the R-loop.^15^ Activation of nuclease domains following R-loop formation results in double-stranded breaks (DSBs) at the target site.^16^^,^^17^^,^^18^ At the site of DSBs, DNA repair would occur via either end-joining mechanisms (non-homologous end-joining or microhomology-mediated end-joining) or homology directed repair (HDR).^19^ The former, being the more efficient pathway, can be used to inhibit disease progression by disrupting the gene with uncontrolled indels, but also carrying the risk of unwanted mutations.^19^ The latter aims to correct the sequence back to a healthy wild-type state by co-delivering a CRISPR-Cas system with an HDR template, applicable for diseases of both recessive and dominant nature.^20^ Nonetheless, HDR remains less efficient in postmitotic cells, limiting its translational applications.^19^ Consequently, *ex vivo* therapies that allow for clonal selection and reinfusion may benefit most from this strategy. Despite these limitations, CRISPR-Cas systems have advanced into multiple clinical trials, which are covered in subsequent sections.

#### Base editors

Base editors, a safer alternative to traditional CRISPR-Cas systems, could correct point mutations without relying on DSBs and HDR, making them suitable for postmitotic cell genomic editing.^21^^,^^22^^,^^23^ Comprising a catalytically inactive Cas nuclease and a deaminase, these editors act on single-stranded DNA (ssDNA) rather than double-stranded DNA (dsDNA) to minimize DSB-associated by-products.^24^ Upon target DNA binding, the gRNA and DNA strand form an "R-loop," enabling the deaminase to modify DNA bases. The inactive nuclease induces a nick in the non-edited DNA strand, prompting repair using the edited strand as a template for efficient eukaryotic cell repair.^66^^,^^67^ To date, three classes of DNA base editors have been identified: adenine base editors (ABEs), cytosine base editors (CBEs), and glycosylase base editors (GBEs). ABEs can convert an A:T base pair into a G:C base pair, while CBEs can convert a C:G base pair into a T:A base pair.^24^^,^^25^^,^^26^ As for GBEs, they can conduct C:G to G:C transversions.^27^^,^^28^ Together, ABEs, CBEs, and GBEs accounts for six of 12 possible changes (A to G, C to T, C to G, G to C, T to C, and G to A). Alternatively, Cas13-guided base editing of adenosine-to-inosine or cytosine-to-uracil have also been achieved in RNA to rescue disease-relevant sequences and yielding functional protein.^29^ This RNA modification is non-permanent and reversible, rendering it relatively safer for potential *in vivo* therapeutic application.

#### Prime editing

Prime editing, pioneered by Anzalone et al.*,*^30^ enhances base editing capabilities, enabling all 12 possible modifications.^31^ Besides base substitution, prime editing allows rewriting of up to 48 nucleotides at a specific locus of interest, enabling precision editing of frameshift mutations through insertions and deletions.^32^ The prime editor complex comprises a Cas9 nickase and a modified reverse transcriptase (RT) along with a multifunctional prime editing gRNA (pegRNA). Utilizing a similar nickase activity to base editing, prime editing provides comparable advantages such as the capacity to target postmitotic cells and avoid the introduction of error-prone DSBs. Mechanistically, the prime editor complex binds to the target region and introduces a nick in the non-complementary DNA strand, three bases upstream of the PAM site. This generates a DNA flap with a 3′ OH group that binds to the primer binding site of the RNA template, serving as a primer for the RT. The RT extends the 3′ flap using pegRNA as a template and FEN1’s endonuclease activity promotes hybridization with the complementary strand to complete the editing process.^30^

#### CRISPR-associated transposons

CRISPR-associated transposons (CASTs) are another new addition to the CRISPR family. It constitutes the co-option of a diverse range of CRISPR-Cas systems (Type I, I-F, I-B, V-K) by Tn-7-like transposons to mediate RNA-guided DNA insertion.^33^ Despite their notable diversity, these systems share key components: CRISPR effector (Cas12k or Cascade); CRISPR array; transposition machinery consisting of TniQ and TnsC accompanied by a transposase (TnsB or TnsA-TnsB); cargo DNA flanked by transposon left end and right end elements.^34^ The DNA-targeting effectors, guided by CRISPR-RNAs (crRNAs) derived from the CRISPR array, along with *trans*-activating RNA (tracrRNA) or a single-molecule gRNA for CRISPR effectors, recognize specific target sites with the required 5′-GTN PAM and bind to them.^33^ The formation of a complete R-loop structure within Cas12k is achieved through the recruitment of TniQ and TnsC, serving as a structural checkpoint for the assembly of the transposon recruitment complex. Mechanistically, the CRISPR effector recruits transposase components, with TniQ playing a crucial role in bridging the RNA-bound CRISPR effector and the ATP-dependent TnsC filament on the target DNA. TnsC, identified as an AAA+ ATPase, actively forms a hexameric ring in association with the double-stranded DNA, to establish the insertion site for the transposon DNA at a predetermined base pair downstream of the target site.^33^^,^^35^ The programmable DNA-integration machinery of CASTs has been leveraged on to enable targeted insertion of large DNA payloads (>10 kb) within certain prokaryotic genomes, again bypassing the need for DSBs and HDR.^36^^,^^37^ Using this capability, the targeted insertion of intact gene copies into the genome could restore protein function, addressing the effects of loss-of-function variants in autosomal recessive disorders. However, CASTs are constrained to introducing normal genes, not replacing them, posing limitations for autosomal dominant disorders driven by gain-of-function variants. Furthermore, CAST applications are currently limited to prokaryotes, necessitating optimization for its genome engineering capabilities in eukaryotic applications.

### Effectors at the RNA level

At the RNA level, two main strategies exist: antisense RNA using short oligonucleotides to alter RNA processing, and mRNA for transient protein expression in the cytoplasm. Notably, RNA-level effectors are smaller than DNA-level counterparts, aiding their packaging into delivery vectors. However, there are still challenges present with introducing RNA, including rapid degradation by ubiquitous RNases, delivery of negatively charged RNA across hydrophobic cell membranes, and potential immunogenicity leading to cell toxicity and compromised protein translation.

#### Antisense oligonucleotides

Antisense oligonucleotides (ASOs) are short (<20 nucleotides in length), synthetic, single-stranded deoxyribonucleotide analogs utilized for gene silencing.^38^ ASOs can utilize diverse mechanisms, but RNA cleavage induction and splicing regulation are the more commonly employed ones among approved ASO drugs.^6^ For cleavage induction, ASO hybridizes with targeted RNA via complementary Watson-Crick base pairing to form an ASO-RNA heteroduplex triggering RNA degradation mediated by RNase H.^39^ Since RNase H is present in both nucleus and cytoplasm, ASOs’ druggable targets could be extended to non-coding elements.^40^^,^^41^ Alternatively, binding of ASOs to RNA can also modulate splicing by preventing the binding of splicing factors through steric hindrance. Pre-mRNA is then prevented from undergoing appropriate splicing, downregulating its protein for disease intervention.^42^^,^^43^^,^^44^ Similarly, ribosomal binding could also be sterically inhibited, leading to translational arrest.^38^ However, given their limited half-life *in vivo* despite chemical modifications to enhance stability and their ability to only diminish gene expression, they are currently applicable only in diseases that require transient reductions in gene expression.^45^

#### RNA interference

RNA interference (RNAi), like ASOs, temporarily silences genes by introducing short duplex RNAs (i.e., small interfering RNAs [siRNAs] and microRNAs [miRs]) to disrupt post-transcriptional gene expression.^46^ In the cytoplasm, siRNAs are generated through Dicer, an endonuclease-mediated processing of double-stranded RNAs (dsRNAs) that are either transcribed within the cell or exogenously introduced. These short duplexes, typically consisting of 21–23 nucleotides with a two-nucleotide 3′ overhang, bind and activate the RNA-induced silencing complex (RISC). Within the RISC, the endonuclease argonaute 2 (AGO2) cleaves the sense strand of the duplex, while the antisense guide strand remains bound to the RISC. The guide strand then directs the active RISC to cleave mRNA that is fully complementary, resulting in targeted gene silencing.^47^^,^^48^^,^^49^ In contrast, miRs could regulate multiple mRNAs simultaneously.^50^ Primary miRs transcribed by RNA polymerase II are processed by the DROSHA-DiGeorge Critical Region 8 (DGCR8) complex, generating pre-miRs.^51^ These pre-miRs are subsequently exported to the cytoplasm and further broken down by Dicer, resulting in functional miRs of 18–25 nucleotides. miR undergoes strand selection, in which one strand is selectively loaded into miR-induced Silencing Complex, while the other is discarded.^46^^,^^52^ With partial complementarity to the 3′ untranslated region of mRNA, miR can target these mRNAs for degradation or translational repression.^53^^,^^54^ On the other hand, miRs could also be silenced by antagomirs.^55^

Preclinically, several miR applications in CVDs exhibit promising therapeutic potential. One of them is miR-99a targeting the mTOR signaling pathway to suppress NLRP3 inflammasome activation and enhance macrophage autophagy, thereby alleviating atherosclerosis.^56^ Moreover, miR-99a has been shown to prevent apoptosis and promote autophagy with demonstrated cardioprotective effects in post-infarction LV remodeling in a murine myocardial infarction model.^57^ Likewise, adenovirus-delivered miR-214 or miR-21 induces improvements in LV remodeling and reductions in myocardial apoptosis in rat models of MI or ischemia-reperfusion injury.^58^^,^^59^ Similarly, positive outcomes are observed in adult porcine models of CVD with anti-miR-92a and anti-miR-15, preventing LV remodeling and reducing infarct size.^60^^,^^61^ Beyond its application in MI, miR has proven effective in addressing prevalent phenotypes of CVD such as cardiac hypertrophy and fibrosis. In the presence of hypertrophic stimuli, the miR-212/132 family is frequently upregulated, exerting a pivotal influence on the regulation of cardiac hypertrophy and autophagy through the FoxO3 transcription factor. Consequently, the inhibition of miR-132 with anti-miR-132 has been demonstrated to effectively rescue cardiac hypertrophy and heart failure, and has since progressed to clinical trials.^62^^,^^63^ Additionally, the inhibition of Jagged1/Notch signaling with locked nucleic acid anti-miR-652 attenuates cardiac hypertrophy, resulting in improved heart function and reduced cardiac fibrosis.^64^ Despite these advancements, challenges, such as differential miRNA expression between men and women impacting treatment outcomes, and the need for a comprehensive understanding of underlying mechanisms, persist in translating miR-based therapeutics into clinical applications for CVDs.^65^

#### Circular RNA

Besides miRNA, circular RNA (circRNA) is also an endogenous non-coding RNA molecule that is involved in gene regulation. CircRNA is formed through back-splicing of pre-mRNA, where the 5′ terminus of an upstream exon is spliced with 3′ terminus of a downstream exon. The distinctive structure of circRNA, a covalently closed loop without a 5′ cap or 3′ polyadenylated tail, addresses RNA stability concerns as its circular configuration enhances resistance to RNase, leading to a half-life at least 2.5 times longer compared with linear counterparts, while still maintaining effective regulation of gene expression as demonstrated by their linear forms.^66^^,^^67^^,^^68^^,^^69^ They can act as miR sponges by binding to and sequestering miRs, thereby inhibiting their interaction with target mRNAs leading to an upregulation of target genes.^70^^,^^71^^,^^72^ Additionally, circRNAs can act as protein decoys by associating with RNA-binding proteins to regulate translation. For example, *circPABPN1* binds to HuR and inhibits its interaction with PABPN1 mRNA, leading to the suppression of PABPN1 translation.^73^ While circRNAs are predominantly non-coding in nature, it has been discovered that certain circRNAs that either possess N6-methyladenosine modifications or harbor internal ribosomal entry sites can undergo translation to produce functional peptides.^74^^,^^75^^,^^76^ Nevertheless, the field of circRNA therapeutics is still in its nascent stages, and there is a need to further optimize the design and circularization efficiency of synthetic circRNA.^77^

#### Direct mRNA delivery

mRNA-based therapeutics harness the inherent cellular processes of transcription and translation to achieve their therapeutic benefits. The mechanism involves the introduction of synthetic mRNA, engineered to resemble natural mRNA but with improved stability and translational efficiency, into target cells. In the design of mRNA drugs, modifications can be performed on structural elements, namely the protein-encoding open reading frame (ORF), the 5′ and 3′ untranslated regions (UTRs), the 5′ cap structure, and the 3′ poly(A) tail.^78^ Optimizing the ORF by incorporating a GC-rich sequence and modifying nucleotides like 5-methylcytidine (m5C) and pseudouridine (Ψ) has been shown to reduce immunogenicity and improve translation efficiency.^79^^,^^80^ Notably, the use of N1-methylpseudouridine (m1Ψ) in mRNA vaccines for SARS-CoV-2 has led to less immunogenic response and significantly increased protein production compared with Ψ-containing mRNAs.^81^ Codon optimization can further enhance translation efficiency.^82^ However, it is essential to carefully consider synonymous codon changes, as they may lead to altered folding pathways that implicate the eventual protein structure and function.^83^ Stability is a key aspect, and this is achieved through incorporation of synthetic 5′ capping using cap analogs like ARCA (anti-reverse cap analog) and 3′ poly(A) tails, which can also enhance translation efficiency.^84^^,^^85^ Additionally, mRNA contains 5′- and 3′-UTRs with secondary structures that play a vital role in ribosome scanning, translation regulation, and mRNA stability.^78^ Modifying these UTRs can extend mRNA half-life and enhance protein translation.

In contrast to DNA-based drugs, mRNAs have higher transfection efficiency as they do not require entry into the nucleus to function.^86^ These mRNAs undergo translation to produce the intended therapeutic protein and degrade shortly afterward. This eliminates any potential risk of insertional mutagenesis, but also necessitates repeated administration to sustain therapeutic levels.^87^^,^^88^ This protein can serve multiple purposes in a transient manner, including the replacement of deficient or dysfunctional proteins, activation of the immune system, or cellular reprogramming by expressing transcription or growth factors.^89^ A recent development involves a modified nucleoside-containing mRNA encoding a chimeric antigen receptor (CAR) designed against fibroblast activation protein (FAP), a marker of activated fibroblasts. Administered via CD5-targeted lipid nanoparticle delivery, these anti-fibrotic CAR T cells, when injected into a mouse heart failure model, demonstrated the ability to restore cardiac function and reduce interstitial fibrosis, highlighting the substantial potential of mRNA-based therapeutics.^90^ Despite this promise, delivering therapeutic mRNA to target cells remains a crucial challenge. The negative charge and susceptibility to RNases make it challenging for mRNA to traverse the hydrophobic cell membrane for protein production.^91^ Therefore, developing precision carriers for mRNA delivery is vital, and various non-viral delivery systems have been explored for this purpose.^92^

### Effectors at the protein level

#### RNA aptamers

RNA aptamers are short synthetic single-stranded oligonucleotides that adopt precise three-dimensional structures upon folding, conferring high affinity and selectivity to various molecular targets such as small molecules, proteins, nucleic acids, and even cells and tissues. These aptamers are synthesized through the systematic evolution of ligands by exponential enrichment (SELEX) process.^93^^,^^94^ In the basic SELEX procedure, a single-stranded RNA library containing randomized sequences of 20–100 nucleotides in length, is exposed to the target. Through iterative rounds of segregating the bound sequences from those that do not, followed by retrieval and amplification of the bound sequences, aptamers exhibiting optimal binding properties gradually become enriched within the library. The SELEX methodology has evolved beyond targeting individual molecules, expanding its scope to various SELEX variants that enhance target specificity. Cell-SELEX utilizes whole living cells as targets and is valuable for isolating aptamers against transmembrane proteins.^95^ However, nonspecific binding could arise due to cell damage and cell death during the process. Cross-Over SELEX and Tissue-SELEX address these issues by combining whole-cell and purified protein targets or by targeting tissue components, respectively.

Functionally, aptamers are versatile, as they can be easily modified and engineered into aptamer-drug conjugates and targeted drug delivery systems, enabling their translation into therapeutic applications.^96^^,^^97^ Recent advancements in therapeutic research showcased their diverse applications, particularly in cancer research. First, akin to monoclonal antibodies, aptamers serve as inhibitors, disrupting target protein function.^98^^,^^99^ Second, their ability to bind specific cell-surface targets facilitates targeted drug delivery, promoting the internalization of drugs, siRNA, small-molecule drugs, and nanoparticles.^100^^,^^101^^,^^102^ Last, aptamers can be easily coupled with liposomes and other carriers, establishing a versatile delivery system for targeted transport of small molecules, peptides, nucleic acids, and even the CRISPR-Cas9 system.^101^^,^^103^ Considering their attributes—ease of generation, cost-effectiveness, minimal batch-to-batch variations, reversible folding, and significantly lower immunogenicity, aided by modified sugars at the 2′-position mitigating Toll-like receptor-mediated immune reactions—aptamers emerge as a preferred and safer alternative to monoclonal antibody therapeutics.^104^ Notably, aptamers like pegaptanib sodium have obtained approval for macular degeneration by inhibiting vascular endothelial growth factor activity, reducing pathological angiogenesis, and are actively under clinical evaluation for diverse indications.^105^

## Cardiac-specific delivery of RNA therapeutics

The broad range of RNA therapeutic modalities described in the previous section demonstrate the versatility and rapid adaptability of easily reprogrammable RNA molecules compared with conventional small-molecule pharmaceutical compounds and drugs. However, the delivery of RNA therapeutics requires additional considerations, primarily due to the inherent instability of unmodified RNA strands through degradation by ubiquitous RNase activities. In addition, ubiquitous expression of certain RNA therapeutics can result in varying effects across different cell types. In the field of cardiovascular regeneration, the inhibition of miR-15 or miR-34 can provide therapeutic benefits by augmenting cardiomyocyte proliferation, regulating cardiac remodeling and the effects of aging.^106^ However, in other cell types, proliferation inhibitors like miR-15 and miR-34 are known tumor suppressors.^107^ There is thus a need to reduce unspecific cellular uptake and off-target effects of RNA therapeutics, potentially through the improvements to tissue-specific delivery methods. The following subsections cover the fundamentals of the delivery systems for RNA therapeutics and the recent advances to enhance cardiac-specific targeting and cytosolic localization.

### Adeno-associated virus delivery vectors

The packaging of RNA therapeutics within viral capsids for long-term gene transfer into tissues and cells has been a subject of enduring research interest. Investigators have focused on the adeno-associated virus (AAV) as a vector, as it has been shown to robustly deliver the packaged genetic material with negligible immune response compared with other viral vectors.^108^ As such, AAV vectors are suitable for the repeated dosing of the RNA therapeutics potentially required for cardiac disease treatment. AAV vectors are also capable of transducing postmitotic cells, making them highly suitable for cardiovascular treatment of terminally differentiated cardiovascular cell types that do not divide.^109^ In addition, while AAV vectors are viral vectors, they exist in the host cell in an extra-chromosomal state and only integrate at a specific loci of the host genome, reducing the possibility of unwanted DNA damage.^109^ One limitation of AAV vectors is the restricted genetic package size of 5.2 kb. Consequently, researchers are actively looking for ways to bypass this limit.^109^

Another limitation of AAV vectors as a delivery medium is their innate liver tropism. Systemic administration of AAV vectors leads to their accumulation in the liver, an inherent problem associated with the filtration function of the liver.^110^ In spite of the liver tropism, studies have reported that several AAV serotypes do exhibit cardiac tropism in animal models, such as AAV6, AAV8, and AAV9, although the results across the different species vary.^108^^,^^111^ These AAV vectors have been utilized to transfer gene expression cassettes to rescue animal models of monogenic cardiac disorders.^112^^,^^113^ Similarly, RNA therapeutics such as miRs have been delivered using cardiotropic AAV vectors in animal models to restore cardiac function or induce regeneration.^114^^,^^115^^,^^116^^,^^117^ To further improve targeting to specific cell types, Muik et al. designed custom AAV vectors with ablated natural capsid proteins while expressing adaptor proteins to allow coupling to ligands that target selectively expressed cell-surface receptors. These custom vectors were demonstrated *in vitro* to only transduce cells expressing the ligand-matching receptors and result in highly expressed tumor targeting *in vivo* with no detectable signal in the lung, liver, spleen, or kidney in mice models.^118^

While steady progress into AAV-mediated tissue targeted genetic material delivery has been achieved, the bar is set high for the translational use of AAV vectors in clinical space. In the CUPID (Calcium Upregulation by Percutaneous Administration of Gene Therapy in Cardiac Disease) phase II clinical trial, safety was demonstrated in advanced heart failure patients receiving AAV1 vectors carrying the SERCA2a gene, a potentially therapeutic gene known to address the deficiency associated with heart failure. However, the gene therapy did not lead to an improvement in the clinical course of advanced heart failure patients compared with the placebo treatment, attributed to the lower-than-expected uptake of the AAV1 vector.^119^ Interestingly, a follow-up analysis conducted at the 3-year mark demonstrated comparably better overall cardiac health in the high-dosage treatment group, suggesting that improvements to cardiac tropism and consequently increased expression of the therapeutic gene were necessary for favorable clinical trial results.^120^

Pla et al. notably demonstrated recently an alternative novel usage of AAV vectors for gene delivery. To improve heart transplantation outcomes, the authors demonstrated that gene therapy could be administered *ex vivo* by perfusing donor hearts with specific AAV serotypes pre-operatively. These donor hearts were shown to achieve robust transgene expression post heterotypic transplant into hosts, with no off-target gene or protein expression detected in the host native heart or noncardiac tissues.^121^ Therefore, AAV vector transduction could be taken advantage of in a transplantation setting to achieve tissue-specific transgene expression.

### Nanoparticle delivery vehicles

Lipid nanoparticles (LNPs) are the only delivery vehicles for RNA therapeutics that are clinically approved and were the choice of RNA delivery platform for the mRNA vaccines against COVID-19.^122^^,^^123^ Typical LNPs are composed of ionizable lipids, cholesterols, phospholipids, and a polyethylene glycol (PEG) lipid.^124^^,^^125^^,^^126^ Ionizable lipids are designed to be positively charged during production of LNPs to maximize interactions with nucleic acids to increase their loading efficiency, while remaining mostly neutral during delivery in systemic circulation to avoid sequestration by immune cells. Most importantly, upon entering the cells through endocytosis, these ionizable lipids acquire positive charges again in the acidic environments of the endosomes, which destabilizes the endosomal membrane. Entrapment within the endosome and eventual accumulation and degradation within the lysosomes is the main limitation preventing therapeutic cargo from reaching the cytosol or other desired destinations within the cell.^124^ The inclusion of ionizable lipids and helper lipids (such as DOPE) to aid in destabilizing the endosomal membrane contributes to the occurrence of endosomal escape (EE), but the topic of EE is highly complex and currently not completely understood.^127^

Initial work revealed that only 2%–3% of LNP-complexed siRNA underwent EE, but experimentation with changes to the ionizable lipid component has improved EE ratios, defined as copies of the cargo outside of the endosomes as a ratio to total cargo taken up within the cell.^128^^,^^129^ Sabnis et al. showed that while LNPs containing MC3 amino lipid demonstrated better *in vitro* uptake of the LNPs, the resultant amount of cytosolic mRNA detected per cell was much lower (EE: 2.5%), compared with Lipid 5-based LNPs, which had a lower LNP uptake but with a much higher cytosolic mRNA detected per cell (EE: 15%). The topic of EE is of much research interest as improvements to the EE ratio allows for a reduction in therapeutic dosage and potential associated toxicity. A recently published review extensively covers the usage of peptides and proteins to enhance EE in nanoparticle delivery systems.^130^ The authors concluded that the use of peptide motifs such as the cell-penetrating peptide does not enhance EE even in permissive culture conditions. In contrast, EE with the help of pore-forming proteins or phospholipases are promising, requiring a relatively low concentration of cargo (in this case, 60 pM DNA) *in vitro* to effect gene expressions.^131^ However, the delivery of proteins in conjunction with RNA therapeutics has its own set of downsides, particularly the instability of protein structures and increased immunogenicity.^130^

In a similar vein, polymeric nanoparticles (PNPs) can also function as delivery vehicles for RNA therapeutics and other forms of cargo. While LNPs are composed of organic lipid moieties, polymeric nanoparticles are composed of biodegradable or non-biodegradable polymeric materials that can be cationic (for example, polyethyleneimine, polyamidoamine, cationic polyacrylates), non-cationic (PEG, polyesters), or polymers that can respond to environmental stimuli to change their biophysical properties. Jiang et al. reviewed the recent advances in the compositions and synthesis of PNPs extensively.^132^ These PNPs are promising therapeutic carriers as the polymeric components are highly customisable to alter their physical properties, such as masking their cationic charges, changing their degradability and bioavailability to reduce toxicity, and as a whole influence how their cargo can be delivered into cells. Currently, PNPs have not achieved Food and Drug Administration (FDA) approval for RNA therapeutic delivery but have been approved for small-molecule drug delivery.^133^

### Cardiovascular-specific uptake through active or passive targeting mechanisms

The delivery systems discussed above to carry the RNA therapeutic cargo have similar considerations for therapeutic usage. The therapeutic cargo must be delivered to the cell type of interest, not result in overt toxicity while in circulation, and be bioavailable in the cytosolic compartments. Systemic administration of AAVs, LNPs, and PNPs will naturally result in the accumulation in various organs and tissues, most notably in the liver.^134^ Therefore, it is necessary to enhance targeted uptake in the tissue types of interest. Rapid uptake into the targeted cells will reduce the half-life of the therapeutics in circulation, potentially reducing their inherent toxicity, and reduce the therapeutic dosage required to overcome the hurdle of EE.

Oral delivery has been suggested as a method to target the gastrointestinal tract, but both *in vitro* and *in vivo* models showed that siRNA-complexed LNPs could not withstand the harsh conditions of the gastrointestinal tract.^135^ On the other hand, some success has been observed in the targeting of the myocardium. Direct intramyocardial injection of LNPs complexed with modified mRNA (modRNA; uridine replaced by pseudouridine) resulted in highly specific expression in both the rat and porcine myocardium with significantly lower expression in other organs such as the liver, spleen, and lung.^136^ Sultana et al. later identified modRNA with uridine replaced by N1-methylpseudouridine-5′-triphosphate transfected using positively charged carrier lipids in a sucrose-citrate buffer to achieve optimal localized protein translation via direct myocardial injection in mice.^137^ Protein expression was observed within minutes and persisted for up to 10 days, which is advantageous for targeted treatment of sudden acute cardiac diseases.

Aside from altering the route of administration to invasive direct myocardial injections, specific cardiovascular targeting through systemic administration can be achieved broadly through two mechanisms: active or passive targeting. Active targeting involves modifying the delivery system with a ligand that has binding interactions with specific biomolecules expressed on the surfaces of cell types of interest. FDA-approved examples are givosiran and lumasiran, which are GalNAc-conjugated siRNA and ASO drugs respectively.^138^^,^^139^ GalNAc binds asialoglycoprotein receptor, highly expressed in the target hepatocyte cells and absent in other cell types, allowing for preferential endocytosis of the drug molecules. In the context of LNPs, Kedmi et al. demonstrated that membrane-anchored lipoprotein could be incorporated in their siRNA-loaded LNPs to take advantage of targeting specificity of monoclonal antibodies. The membrane-anchored lipoproteins contain an interchangeable monoclonal antibody domain for specific uptake of LNPs by their targeted leukocyte subsets *in vivo*.^140^ Similar strategies to modify the surface of LNPs was demonstrated by the same group to target cancer cells through antibody affinity, as well as the gut cells through the binding affinity of α4β7 integrin found in the gut.^141^^,^^142^ Currently, no LNP modifications have been published with increased affinity for the heart. However, a number of peptide sequences with binding affinity for cardiomyocytes have been identified, such as CTP (from M13 phage),^143^^,^^144^ PCM1 (from filamentous phage),^145^ and AT1-binding ligand.^146^ Therefore, these peptide sequences may be of interest to enhance cardiovascular active targeting on LNPs.

On the other hand, passive targeting is the preferential targeting of the delivery systems to specific organs and cell types without the need for antibody fragments, peptides, or other ligands that bind cell-surface receptors. The targeting is achieved by altering the inherent characteristics of the nanoparticles, such as their sizes, surface charges, and protein coronas.^147^ For example, smaller, negatively charged LNPs translocate more efficiently and deeper into the lymph nodes.^147^ 7C1, a low molecular weight polyamine and lipid formulation preferentially delivers siRNA to endothelial cells *in vivo* when administered intravenously.^148^ However, in comparison with active targeting strategies, the molecular reasoning behind why passive targeting strategies work remains unclear. A recently developed polylipoic acid PNP by Castellani et al. termed F127@PLA-NP was shown to preferentially accumulate in the myocardium when delivered intravenously in mice due to passive targeting.^149^ Interestingly, the antioxidant properties of the lipoic acid were demonstrated to exhibit cardioprotection against post-ischemia reperfusion *in vitro*. Despite these findings, the authors were unable to elucidate the underlying mechanism resulting in the myocardial accumulation.

While the search for active targeting strategies starts with a hypothetical modification that enhances targeting due to known interactions with cell types of interest, passive targeting strategies are better uncovered by systematically testing various modifications and molar ratios. Cheng et al. developed the novel strategy of selective organ targeting (SORT) LNPs, which were traditional LNPs modified with an additional supplemental component, termed SORT lipid (cationic, anionic, or uncharged).^150^ By systematically testing various modifications of the SORT LNP components including the SORT lipid, they demonstrated tissue-specific targeting of the lung, spleen, or liver when injected intravenously. Similarly, Ni et al. systematically showed that utilizing specific types of piperazine-derived lipids, in combination with specific molar ratios for the individual components of LNPs, can deliver cargo preferentially to non-hepatocytes *in vivo*, most notably to liver and spleen immune cells at low dosage (0.3 mg/kg).^151^

Currently, systematic research identifying passive targeting strategies to the heart has not been done, but there is sufficient interest in the use of various nanoparticles to treat CVDs and provision of cardioprotective effects.^152^ Interestingly, passive targeting of liposomes and micelles was demonstrated to have prolonged retention and specific accumulation in infarcted regions of the heart.^153^ Therefore, there is room for discovering other passive targeting strategies to promote cardiovascular targeting in general. It is also promising to utilize both passive and active targeting strategies in tandem to enhance delivery directly to the heart.

### Extracellular vesicle-mediated therapeutic strategies

In contrast to the exogenously synthesized therapeutic delivery vehicles previously discussed, extracellular vesicles (EVs) are nanoscale particles that also carry a variety of cargo (proteins, lipids, RNA, DNA) encapsulated within a membrane assembled endogenously within host cells. While these endogenous nanoparticles are traditionally studied as a biological tool for cell-cell communication and interaction across extracellular space, there has been increasing interest in utilizing EVs for therapeutic delivery.^154^^,^^155^^,^^156^^,^^157^ EVs are broadly categorized based on their sizes, of which the smallest 50- to 150-nm particles termed exosomes and 100- to 1,000-nm particles termed microvesicles have been utilized as unique carriers of therapeutics.^158^ In addition to the differences in sizes, EVs also differ in how they are released from cells, the ratios of the diverse cargos enriched within them, as well as their cellular source.

Endogenously generated EVs have been reported to contain a range of mRNAs, mature miR, as well as a whole range of ncRNA sequences such as ribosomal RNAs, long non-coding RNAs (lncRNAs), PIWI-interacting RNAs, and transfer RNAs. This transcriptomic heterogeneity within EVs limits their therapeutic applications with our inability to fully characterize EV populations. In addition, the heterogeneity extends to the varied presence of other lipids, proteins, and DNA within the EVs, and EV preparations used for preclinical work are considered as the secretome, as they exist as a mixture with other extracellular elements.^159^^,^^160^ Consequently, these endogenously generated EVs play a different role as an RNA therapeutic vehicle compared with the exogenously synthesized vehicles, as they cannot be synthesized containing a completely controlled and defined composition, much less a single targeting RNA therapeutic. However, similar to LNPs, EV uptake into recipient cells is primarily through endocytosis, which leads to the same issue of poor EE ratios resulting in suboptimal release of therapeutic cargo into the intended cell compartments.^161^ Therefore, research to improve EE specifically for EVs is ongoing to enable more efficient use of EVs for therapeutic purposes.^162^

The heterogeneous populations of EVs also have therapeutic advantage. Researchers have shown that delivering EVs from an appropriate source and type can result in disease-specific amelioration in the various organs.^160^ Particularly of interest are exosomes derived from mesenchymal stem cells (MSC-EVs), which have been shown to enhance rejuvenation and have anti-aging properties across several cell types.^163^^,^^164^^,^^165^ Specifically for the heart, MSC-EVs have been demonstrated to aid in myocardial repair, with cardioprotective effects and improvements to ejection fraction conferred to the post-ischemia heart.^166^^,^^167^^,^^168^ The improvement to heart function was markedly improved when the MSC-EVs were loaded with macrophage migration inhibitory factor (MIF) from the donor MSCs, by overexpressing MIF.^167^ This approach confirms that EVs can be selectively enriched for certain cargo by modifications to the donor cell source, potentially making them comparable to exogenously synthesized vehicles.

Another primary advantage of these endogenously generated EVs is their comparably reduced immunogenicity, being a natural component of extracellular space. EVs can also be harvested from autologous sources, making them immune neutral,^160^ or from commonly used proliferative cell types such as MSCs, red blood cells,^169^ and HEK293 cells,^170^ human plasma,^171^ or plant materials.^172^ EV preparations from these donor sources can be modified for the loading of specific cargo, such as the enrichment of potential RNA therapeutics, through genetic engineering. Despite this diversity, MSC-EVs are still the closest to therapeutic usage, with MSC-EVs as secretomes already being tested in clinical trials for cardioprotective purposes (NCT05669144). MSC-EVs loaded with RNA therapeutics have not been utilized for cardiovascular health, but miR-loaded MSC-EVs are being tested for use in treating ischemic stroke (NCT03384433) and against pancreatic cancer (NCT03608631). Therefore, EVs are a promising alternative delivery vehicle for RNA therapeutics, with naturally reduced immunogenicity and cardioprotective capabilities.

## Therapeutic targets in CVDs

The RNA therapeutics described in the previous sections are broadly able to effect permanent genomic changes as well as transiently alter their transcribed counterparts. Therefore, it is appropriate to discuss the potential therapeutics suitable to treat the various CVDs, which encompass a range of Mendelian or acquired disorders affecting the myocardium, vasculature, and cardiac electrical system.^173^ While Mendelian CVDs, caused by mutations in a single gene, are relatively uncommon, the majority of CVDs are polygenic and are acquired through a complex interplay of genetic and environmental factors.^174^ Deeper understanding of the underlying factors behind the different forms of CVDs will unlock the potential for targeted interventions at the gene, transcript, and protein levels through RNA therapeutics, paving the way for personalized medicine.

### Mendelian CVDs

Mendelian or monogenic CVD is a distinct group of cardiovascular conditions that is caused by a deleterious mutation in a single gene.^175^ These genetic abnormalities can affect various aspects of cardiovascular function, including the heart structure, electrical signaling, and metabolism resulting in cardiomyopathies (i.e., hypertrophic, familial, and transthyretin cardiomyopathies), arrhythmias (i.e., long QT syndrome, Brugada syndrome), connective tissue disorders (i.e., Marfan syndrome, Ehlers-Danlos syndrome, Loeys-Dietz syndrome), and familial hypercholesterolemia. These conditions are estimated to have a combined prevalence of 1.7% in the general population and contribute substantially to morbidity and mortality, particularly among young individuals.^176^ In this context, we will delve into the genetic variants associated with commonly inherited CVDs, specifically hypertrophic cardiomyopathy, familial dilated cardiomyopathy, atrial fibrillation (AF), and familial hypercholesterolemia. Table 1 lists the most common genetic variants associated with these CVDs, which can be generalized to other disorders with similar underlying factors.

**Table 1:** Potential targetable genetic variants associated with commonly inherited cardiovascular diseases

Mendelian Cardiovascular Disease				
Disease phenotype	Gene affected	Common variants	Inheritance pattern	Reference
Hypertrophic Cardiomyopathy	*ACTC1*	Missense mutation: c.496C>G (p.Pro166Ala); c.997G>C (p.Ala333Pro)	Autosomal Dominant	Olson et al.^283^
	*ALPK3*	Nonsense mutation: c.3792G>A (p.Trp1264Ter); c.3781C>T (p.Arg1261Ter); c.33G>A (p.Trp11Ter); c.2812C>T (p.Gln938Ter);	Autosomal Recessive	Phelan et al., Almomani et al., Al Senaidi et al., and Herkert et al.^284^^,^^285^^,^^286^^,^^287^
		Frameshift mutation: c.926_927del (p.Lys309ArgfsTer12); c.1501del (p.Ser501fs)		Jaouadi et al. and Papadopoulos et al.^288^^,^^289^
	*MYBPC3*	Nonsense mutation: c.3811C>T (p.Arg1271Ter); c.2827C>T(p.Arg943Ter); c.2905C>T (p.Gln969Ter); c.2747G>A (p.Trp916Ter)	Autosomal Dominant	Olivotto et al. and Cirino et al.^290^^,^^291^
		Frameshift mutation: c.2373dup (p.Trp792fs); c.927-9G>A		Cirino et al. and Niimura et al.^291^^,^^292^
		Missense mutation: c.772G>A (p.Glu258Lys); c.772G>A (p.Glu258Lys)		Cirino et al. and Niimura et al.^291^^,^^292^
		Missense mutation: c.1469G>T (p.Gly490Val)	Autosomal Recessive	Wang et al.^293^
	*MYH7*	Missense mutation: c.2770G>A (p.Glu924Lys); c.2459C>A (p.Ala820Asp); c.5135G>A (p.Arg1712Gln); c.4130C>T (p.Thr1377Met); c.4066G>A (p.Glu1356Lys); c.3158G>A (p.Arg1053Gln); c.2722C>G (p.Leu908Val); c.2717A>G (p.Asp906Gly); c.2711G>A (p.Arg904His); c.2710C>T (p.Arg904Cys); c.2681A>G (p.Glu894Gly); c.2609G>A (p.Arg870His); c.2539A>G (p.Lys847Glu); c.2513C>T (p.Pro838Leu); c.2221G>T (p.Gly741Trp); c.2221G>C (p.Gly741Arg); c.2207T>C (p.Ile736Thr); c.2167C>G (p.Arg723Gly); c.2167C>T (p.Arg723Cys); c.2156G>A (p.Arg719Gln); c.2155C>T (p.Arg719Trp); c.2146G>A (p.Gly716Arg); c.1988G>A (p.Arg663His); c.1750G>C (p.Gly584Arg); c.1594T>C (p.Ser532Pro); c.1357C>A (p.Arg453Ser); c.1357C>T (p.Arg453Cys); c.1208G>A (p.Arg403Gln); c.1207C>T (p.Arg403Trp); c.788T>C (p.Ile263Thr)	Autosomal Dominant	Cirino et al., Watkins et al., Okada et al., Marsili et al., Wang et al., Vepsäläinen et al., Nykamp et al., Ross et al., Volkmann et al., Marsiglia et al., Gruver et al., Kelly et al., Ko et al., and Tesson et al.^291^^,^^294^^,^^295^^,^^296^^,^^297^^,^^298^^,^^299^^,^^300^^,^^301^^,^^302^^,^^303^^,^^304^^,^^305^^,^^306^
	*MYL2*	Missense mutation: c.173G>A (p.Arg58Gln)	Autosomal Dominant	Richard et al., Flavigny et al., Kabaeva et al., Morner et al., and Lopes et al.^189^^,^^307^^,^^308^^,^^309^^,^^310^
	*MYL3*	Missense mutation: c.281G>A (p.Arg94His); c.445A>G (p.Met149Val); c.281G>A (p.Arg94His)	Autosomal Dominant	Poetter et al., Alfares et al., Nomura et al., Fokstuen et al., and Arad et al.^193^^,^^311^^,^^312^^,^^313^^,^^314^
		Missense mutation: c.170C>A (p.Ala57Asp)	Autosomal Recessive	Osborn et al.^315^
	*PRKAG2*	Missense mutation: c.1453A>G (p.Lys485Glu)	Autosomal Dominant	Liu et al.^316^
	*TPM1*	Missense mutation: c.523G>A (p.Asp175Asn)	Autosomal Dominant	Watkins et al.^317^
	*TNNI3*	Missense mutation: c.616A>C (p.Lys206Gln); c.592C>G (p.Leu198Val); c.485G>A (p.Arg162Gln); c.485G>C (p.Arg162Pro)	Autosomal Dominant	Kimura et al., Rani et al., Mogensen et al., and Doolan et al.^191^^,^^318^^,^^319^^,^^320^
	*TNNT2*	Frameshift mutation: c.508_510GAG^3^ (p.Glu173del)	Autosomal Dominant	Torricelli et al.^321^
		Splice site mutation: c.851 + 1G>A		Thierfelder et al.^190^
		Missense mutation: c.304C>T (p.Arg102Trp); c.418C>T (p.Arg140Cys); c.358T>A (p.Phe120Ile); c.266T>A (p.Ile89Asn); c.305G>A (p.Arg102Gln)		Thierfelder et al. and Fujita et al.^190^^,^^322^
		Missense mutation: c.77C>T (p.Ser179Phe)	Autosomal Recessive	Ho et al.^323^
Dilated Cardiomyopathy	*BAG3*	Frameshift mutation: g.119672256_119677283del; c.727del (p.His243Ter)	Autosomal Dominant	Toro et al. and Franaszczyk et al.^324^^,^^325^
		Nonsense mutation: c.925C>T (p.Arg309Ter)	Autosomal Dominant	Chami et al.^326^
	*FLNC*	Splice site mutations: c.7251 + 1G>A; c.3791-1G>A	Autosomal Dominant	Ortiz-Genga et al.^327^
		Nonsense mutations: c.6976C>T (p.Arg2326Ter)		Ortiz-Genga et al.^327^
	*LMNA*	Nonsense mutations: c.673C>T (p.Arg225Ter); c.961C>T (p.Arg321Ter); c.1063C>T (p.Gln355Ter); c.1294C>T (p.Gln432Ter)	Autosomal Dominant	Jakobs et al. and Møller et al.^328^^,^^329^
	*MYH7*	Missense mutation: c.2513C>T (p.Pro838Leu); c.5635A>G (p.Lys1879Glu); c.5754C > R (p.Asn1918Lys)	Autosomal Dominant	Vasilescu et al., Sajid et al. and van der Linde et al.^330^^,^^331^^,^^332^
	*MYL3*	Nonsense mutation: c.106G>T (p.Glu36Ter)	Autosomal Recessive	Osborn et al.^315^
	*RBM20*	Nonsense mutation: c.2062C>T (p.Arg688Ter)	Autosomal Dominant	van der Linde et al.^332^
	*SCN5A*	Missense mutation: c.2440C>T (p.Arg814Trp); c.5321T>C (p.Phe1774Ser)	Autosomal Dominant	Waldmüller et al. and Olson et al.^333^^,^^334^
		Frameshift mutation: c.2548_2549GT^3^ (p.Phe851fs)		Olson et al.^334^
	*TNNI3*	Missense mutation: c.4C>T (p.Ala2Val)	Autosomal Recessive	Murphy et al.^335^
	*TNNT2*	Nonsense mutation: c.650_652AGA (p.Lys220del)	Autosomal Dominant	Otten et al.^336^
	*TTN*	Nonsense mutation: c.98506C>T (p.Arg32836Ter); c.61876C>T (p.Arg20626Ter); c.67495C>T (p.Arg22499Ter); c.62217T>A (p.Tyr20739Ter); c.85969A>T (p.Lys28657Ter); c.84819G>A (p.Trp28273Ter); c.54638G>A (p.Trp18213Ter); c.85768C>T (p.Arg28590Ter); c.61495C>T (p.Arg20499Ter); c.71980_71986delinsTA (p.Ala23994_Glu23996delinsTer); c.101227C>T (p.Arg33743Ter); c.48283C>T (p.Arg16095Ter); c.70978C>T (p.Arg23660Ter); c.88422G>A (p.Trp29474Ter); c.45322C>T (p.Arg15108Ter); c.41641C>T (p.Arg13881Ter); c.93166C>T (p.Arg31056Ter); c.63025C>T (p.Arg21009Ter)	Autosomal Dominant	Merlo et al., Herman et al., Norton et al. Akinrinade et al., and Felkin et al.^337^^,^^338^^,^^339^^,^^340^^,^^341^
		Frameshift mutation: c.98299_98300del (p.Arg32767fs)		Herman et al.^338^
		Missense mutation: c.95083G>A (p.Gly31695Arg)		Franaszczyk et al.^342^
		Splice site mutation: c.86821 + 2T>A		Norton et al.^339^
Atrial Fibrillation	*CORIN*	Frameshift mutation: c.684dup (p.Met229fs)	Autosomal Dominant	
	*GJA5*	Missense mutation: c.685C>A (p.Leu229Met); c.661C>A (p.Leu221Ile); c.262C>T (p.Pro88Ser); c.253G>A (p.Val85Ile); c.4780G>A (p.Asp1594Asn)	Autosomal Dominant	Sun et al. and Gollob et al.^343^^,^^344^
		Nonsense mutation: c.145C>T (p.Gln49Ter)	Autosomal Dominant	Yang et al.^345^
	*KCN1B*	Missense mutation: c.254G>A (p.Arg85His); c.363C>G (p.Cys121Trp)	Autosomal Dominant	
	*KCNA5*	Missense mutation: c.143A>G (p.Glu48Gly); c.1828G>A (p.Glu610Lys)	Autosomal Dominant	Olson et al.^346^
		Nonsense mutation: c.1123G>T (p.Glu375Ter)	Autosomal Dominant	Christophersen et al.^347^
	*KCNJ2*	Missense mutation: c.199C>T (p.Arg67Trp); c.653G>A (p.Arg218Gln)	Autosomal Dominant	
	*KCNQ1*	Missense mutation: c.418A>G (p.Ser140Gly); c.520C>T (p.Arg174Cys); c.521G>A (p.Arg174His); c.604G>A (p.Asp202Asn); c.674C>T (p.Ser225Leu); c.692G>A (p.Arg231His); c.797T>C (p.Leu266Pro); c.805G>A (p.Gly269Ser); c.815G>A (p.Gly272Asp); c.1615C>T (p.Arg539Trp); c.1664G>A (p.Arg555His); c.1697C>T (p.Ser566Phe); c.1702G>A (p.Gly568Arg)	Autosomal Dominant	Richards et al.^348^
		Nonsense mutation: c.513C>G (p.Tyr171Ter); c.914G>A (p.Trp305Ter); c.1075C>T (p.Gln359Ter); c.1588C>T (p.Gln530Ter)	Autosomal Dominant	
		Frameshift deletion: c.200_210del (p.Pro67fs); c.796del (p.Leu266fs); c.998_999del (p.Ser333fs); c.1014CTT^1^ (p.Phe340del); c.1265dup (p.Phe423fs); c.1343dup (p.Glu449fs); c.1892_1911del (p.Pro631fs)	Autosomal Dominant	
		Splice site mutation: c.921 + 1G>T	Autosomal Dominant	
	*MYL4*	Missense mutation: c.31G>A (p.Glu11Lys)	Autosomal Dominant	Orr et al.^207^
		Nonsense mutation: c.361C>T (p.Gln121Ter); c.532C>T (p.Gln178Ter)	Autosomal Dominant	Gudbjartsson et al.^349^
		Frameshift mutation: c.234del (p.Cys78fs)	Autosomal Dominant	Gudbjartsson et al.^349^
	*NAPPA*	Frameshift mutation: c.456_∗1del (p.Ter152TrpextTer?)	Autosomal Dominant	Hodgson-Zingman et al.^350^
	*NUP155*	Missense mutation: c.1172G>A (p.Arg391His)	Autosomal Dominant	Zhang et al.^351^
	*SCN3B*	Missense mutation: c.482T>C (p.Met161Thr)	Autosomal Dominant	Olesen et al.^352^
	*SCN4B*	Missense mutation: c.496A>C (p.Ile166Leu); c.485T>G (p.Val162Gly)	Autosomal Dominant	Li et al.^353^
	*SCN5A*	Missense mutation: c.5224G>A (p.Gly1742Arg); c.5126C>T (p.Ser1709Leu); 4219G>A (p.Gly1407Arg); c.3953G>T (p.Gly1318Val); c.2989G>T (p.Ala997Ser); c.1127G>A (p.Arg376His); c.1099C>T (p.Arg367Cys); c.673C>T (p.Arg225Trp); c.665G>A (p.Arg222Gln); c.310C>T (p.Arg104Trp)	Autosomal Dominant	Richards et al.^348^
		Nonsense mutation: c.4864C>T (p.Arg1622Ter)	Autosomal Dominant	
		Frameshift mutation: c.5414_5417del (p.Thr1805fs); c.4844TCT^1^ (p.Phe1616del); c.2550_2551dup (p.Phe851fs)	Autosomal Dominant	
		Splice site mutation: c.4242 + 1G>C	Autosomal Dominant	
Familial Hypercholesterolemia	*APOB*	Missense mutation: c.10580G>A (p.Arg3527Gln)	Autosomal Dominant	Soufi et al.^354^
	*LDLR*	Nonsense mutation: c.97C>T (p.Gln33Ter); :c.337G>T (p.Glu113Ter); c.418G>T (p.Glu140Ter); c.91G>T (p.Glu31Ter)	Semi-Dominant	Loux et al. and Descamps et al.^355^^,^^356^
		Splice site mutation: c.1359-1G>A	Autosomal Dominant	Descamps et al.^356^
		Frameshift mutation: c.518del (p.Cys173fs); c.2447_2450dupAGAA (p.Asn817Lysfs)		Descamps et al.^356^
	*LDLRAP1*	Frameshift mutation: c.71del (p.Gly24AlafsTer32); c.432_433insA (p.Ala145SerfsTer26); c.74dup (p.Gly26TrpfsTer8)	Autosomal Recessive	Garcia et al.^357^
		Nonsense mutation: c.65G>A (p.Trp22Ter); c.406C>T (p.Gln136Ter)		Garcia et al.^357^
	*PCSK9*	Missense mutation: c.185C>A (p.Ala62Asp); c.1399C>G (p.Pro467Ala); c.1069 C > T(p.Arg357Cys); c.1906A > C (p.Ser636Arg)	Autosomal Dominant	Alves et al. and Di Taranto et al.^358^^,^^359^

Please note that this list is not exhaustive, and it only comprises variants classified as pathogenic and definitive for their clinical significance in the ClinVar and ClinGen database, respectively.

#### Hypertrophic cardiomyopathy

Hypertrophic cardiomyopathy (HCM), which is estimated to affect 1 in 500 individuals and may be even more common based on recent evidence, stands as the most prevalent inherited CVD.^177^^,^^178^ HCM is morphologically characterized by the presence of a hypertrophied, non-dilated left ventricle without the influence of another systemic or cardiac disease that could account for the observed degree of wall thickening.^179^ This is often accompanied with asymmetrical involvement of the basal interventricular septum and preserved or increased ejection fraction.^180^ At the cellular level, cardiac myocytes are hypertrophied, in disarray coupled with the presence of interstitial fibrosis.^180^ A comprehensive summary of the causal genes can be found in the review by Marian and Braunwald.^181^ Originally discovered by Pare et al., the p.Arg403Glu mutation in the sarcomere protein β-myosin heavy chain (*MYH7*) gene thought to be responsible for hereditary cardiovascular dysplasia was subsequently established as the genetic basis of HCM.^182^^,^^183^ In a recent breakthrough, RNA therapeutics targeting this pathogenic variant involving two different genome editing tools—an ABE system and Cas9 nuclease delivered by AAV9—have been assessed.^184^^,^^185^ Employing dual-AAV9 vectors, each containing one-half of an ABE to address the limited packaging capacity of AAV9 (∼4.7 kb) for full-length base editors, this strategy achieved modest genomic editing (∼20%–30%) in cardiovascular tissues, which resulted in a 2-fold longer survival than untreated mice.^184^ Despite lower initial editing efficiency in the atria (∼30%) compared with the ventricles (∼70%), a second AAV injection moderately increased atrial editing with additional bystander effects.^184^ The authors also explored AAV9 delivery of RNA-guided Cas9 nuclease to permanently silence the pathogenic allele.^184^ However, it exhibited dose-dependent toxicities, underscoring the narrow therapeutic window and highlighting the advantages of the ABE system over conventional Cas9.^184^ Chai et al.^185^ adopted a similar approach in a humanized mouse model, reporting ∼30% reduction in pathological variants and functional improvement but with lower atrial editing efficiency. While addressing ventricular hypertrophy and fibrosis through partial removal of pathological variants holds promise, both studies emphasize a chamber-specific decrease in pathogenic variants that may result in untreated persistent atrial disease causing progressive atrial fibrosis and fatal arrhythmia. Additionally, the challenge of natural immunity against AAV, affecting 30%–60% of the human population and significantly escalating with a single AAV vector injection, presents a substantial obstacle to AAV therapy. Several other distinct mutations in sarcomere proteins have also been identified as causal genes for HCM. Accounting for half of the familial HCM cases, *MYH7* and *MYBPC3* are the two most prevalent causal genes.^186^^,^^187^^,^^188^^,^^189^ Other less common casual genes include *ACTC1*, *MYL2*, *MYL3*, *CSRP3, TNNT2*, *TNNI3*, and *TPM1*.^188^^,^^189^^,^^190^^,^^191^^,^^192^^,^^193^^,^^194^

#### Familial dilated cardiomyopathy

Dilated cardiomyopathy (DCM) is characterized by the enlargement and dilation of one or both ventricles, resulting in impaired contractility primarily affecting systolic function. Unlike HCM, DCM is associated with a progressive weakening and thinning of the left ventricle wall, leading to reduced efficiency in blood pumping to meet the body’s physiological needs.^179^^,^^195^ Over time, this can lead to heart failure. The estimated prevalence of dilated cardiomyopathy (DCM) could range from 1:250 to 1:2,500 in the general population.^196^^,^^197^ Approximately 20%–35% of DCM cases are identifiable as familial dilated cardiomyopathy (FDC), while the remaining cases are categorized as idiopathic.^197^ However, it is likely that the actual frequency of familial forms is underestimated due to limited family pedigrees and undiagnosed individuals, which can obscure the underlying genetic cause due to variable expressivity and reduced penetrance of the disease gene.^195^ Around 40% of the cases identified as FDC have a known genetic basis attributed to more than 50 genes, and this number continues to grow as more genes are discovered.^198^ FDC is primarily inherited in an autosomal dominant manner, although there have been reports of autosomal recessive and X-linked forms as well.^196^^,^^197^

#### Atrial fibrillation

Atrial fibrillation (AF) is the most common clinical arrythmia, affecting 60 million individuals worldwide.^199^ Similar to prevalent CVDs like hypertension and myocardial infarction, disease pathogenesis of AF is a multifaceted condition influenced by both environmental and genetic factors. Remarkably, about 30% of AF cases, termed Lone AF, manifest without identified cardiac pathology or known risk factors.^200^ Recent studies observe familial aggregation in Lone AF cases, suggesting a heritability of AF, which was particularly evident in the Icelandic population.^201^ A study involving monozygotic twins estimates the heritability of AF to be as high as 62%, indicating a substantial genetic contribution.^202^ Consistent findings also indicate an increased AF risk, particularly with affected first-degree family members or among those with early-onset arrhythmia.^203^ Linkage analysis has identified several causative mutations associated with familial AF, including *KCNQ1, NPPA*, and *TBX5*.^204^^,^^205^^,^^206^ These mutations result in alterations in ion channel function, increased atrial natriuretic peptide levels, and heart malformation, respectively, increasing the susceptibility to AF. In a large-scale study of Icelanders, autosomal recessive mutations in *MYL4* were identified in individuals with early-onset AF.^207^ Furthermore, a whole-genome sequencing analysis involving 14,255 AF cases and 374,939 controls from the Icelandic population revealed a low-frequency missense mutation in the *PLEC* gene associated with increased AF risk.^208^ In recent years, multiple studies also identified loss-of-function (LOF) mutations in the *TTN* gene among individuals with AF, particularly those with early-onset AF.^209^^,^^210^ These findings were further validated in the general population with AF using exome sequencing data from the UK Biobank, emphasizing the strong association between TTN LOF variation and AF, with increased penetrance among individuals with a higher polygenic risk for AF.^211^

#### Familial hypercholesterolemia

Familial hypercholesterolemia (FH) is a genetic disorder affecting 1 in 311 individuals in the general population that can be inherited in either an autosomal dominant or autosomal recessive form.^212^^,^^213^ These individuals are subjected to an increased risk of premature atherosclerosis and cardiovascular complications due to prolonged elevation levels of low-density lipoprotein cholesterol (LDL) in the blood.^213^ If left untreated, individuals with heterozygous FH usually experience coronary heart disease (CHD) before the age of 55 in men and 60 in women.^214^ For individuals with homozygous FH, they tend to develop CHD before the age of 20 and have a shortened lifespan.^215^ The majority (79%) of FH cases are attributed to mutations in the LDL receptor (LDLR) gene, leading to abnormalities in LDLR synthesis, assembly, transport, recycling, or impaired LDL endocytosis. Apolipoprotein B (*APOB*) facilitates the binding of LDL to LDLR, while proprotein convertase subtilisin/kexin type 9 (PCSK9) degrades LDLR. Mutations in *APOB* and *PCSK9* account for 5% and <1% of FH cases, respectively. The remaining 15% of FH cases can be either polygenic or caused by rare mutations in genes such as *APOE*, *SREBP2*, and *STAP1*.^216^^,^^217^ Additionally, there is an extremely rare recessive form of FH caused by a mutation in the *LDLRAP1* gene.^218^

### Acquired CVDs

The Mendelian CVDs previously discussed are well understood, can potentially be treated with monogenic changes through RNA therapeutics, and are unfortunately not the norm for most CVDs. Most cases of CVD have a multifactorial origin, making it difficult to predict the onset, progression, or severity of the disease solely based on an individual’s genetic makeup or environmental exposures. Disease development arises from interactions between initial genetic conditions and exposures to environmental agents (e.g., exercise, stress, smoking) over time and space. These environmental factors may act as compensatory or exacerbating factors in disease development. Over time, these interactions are integrated by dynamic regulatory networks beyond the genomic level, leading to a diverse range of phenotypes at a particular point in time.^219^ However, it is essential to consider that family history of CVD does play a role in modifying future CVD risk, depending on the number and age of affected first-degree relatives. Siblings of CVD patients have approximately a 40% increased risk, while offspring of parents with premature CVD face a 60%–75% increase in risk.^220^ Given the significant impact of genetic predisposition, RNA therapeutics have the potential to intervene and potentially reduce the risk of developing CVD. This section covers the common genetic risk factors associated with CVD that could potentially be targeted by RNA therapeutics. Additionally, we introduce an emerging class of RNA molecules, specifically lncRNAs, that has been widely implicated in CVDs, offering potential targets for diverse RNA therapeutic approaches.

#### Dyslipidemia

Dyslipidemia refers to elevated levels of serum total cholesterol, low-density lipoprotein cholesterol (LDL-C), triglycerides (TG), or decreased serum high-density lipoprotein cholesterol (HDL-C).^221^ Beyond FH, dyslipidemia, considering other forms of lipid abnormalities, is a well-established risk factor for CVD.^221^ Although dietary intake is expected to impact circulatory lipids, plasma levels of lipid species are found to be heritable, indicating a significant role of endogenous regulation in lipid metabolism.^222^ In a study, 35 lipid-species-associated loci have been identified, 10 of which were associated with at least one CVD.^222^ These included novel associations, such as *COL5A1* with cerebrovascular disease, *GALNT16* with angina, *MBOAT7* with venous thromboembolism, *GLTPD2* with atherosclerosis, and *SPTLC3* with intracerebral hemorrhage.^222^ In the case of the most prevalent form of acquired CVD, coronary artery disease (CAD), approximately 20% of known small nucleotide polymorphisms (SNPs) associated with it are also located near genes involved in lipid regulation.^223^ Common variants in genes associated with LDL-C, TG levels, and HDL-C have also been linked to CAD risk.^224^ These findings underscore the importance of genetic variants involved in lipid regulation in the development of CVD.

#### Hypertension

Hypertension, also known as high blood pressure (BP), is another well-established risk factor for CVD.^225^^,^^226^ About 54% of strokes and 47% of CAD worldwide are linked to high BP.^227^ Therefore, genetic variants associated with elevated BP and hypertension are likely to overlap with those implicated in the development of CVD.^228^ Notably, genes like *MTHFR*, *NPPA*, *TBX1*, *TBX5*, *KCNMA1*, and *ENPEP* are of special interest as they are linked to both hypertension and CVD.^228^ Co-localizations of genes in certain regions, such as *AGT-RYR2* on chromosome *1q43*, have been identified as potential "hot spots" for significant candidates related to both BP regulation and heart functioning.^228^ These interconnecting genetic factors between hypertension and CVD have been covered extensively by Kraja et al.^228^ Furthermore, certain genetic variants located in specific loci that are associated with BP regulation (e.g., *SH2B3* and *ZC3HC1*) have been linked to an increased risk of developing CAD.^224^^,^^229^^,^^230^ GWAS studies have also identified SNPs associated with hypertension. In particular, two genetic loci, *NOS3* and *GUCY1A1,* encode for proteins that regulate vascular tone and inhibit atherosclerosis through nitric oxide-soluble guanylate cyclase-cGMP signaling.^224^^,^^231^ Hence, LOF mutations in *NOS3* or *GUCY1A1* genes are linked to higher risks of hypertension and CAD, while inactivating mutations in *GUCY1A1* are associated with an increased risk of peripheral CAD.^232^^,^^233^ Additionally, SNPs in *PDE3A, PDE5A,* and *MRVI1* genes, involved in the NO signaling pathway, have also been associated with CAD, although the precise mechanisms remain unclear.^224^^,^^230^^,^^233^^,^^234^

#### LncRNAs as therapeutic targets

LncRNAs, a subset of non-coding RNAs, are a heterogeneous group of RNAs exceeding 200 nucleotides and are pivotal regulators of gene expression influencing epigenetic, transcriptional, post-transcriptional, translational, and post-translational processes through direct or indirect interactions with DNA, RNA, and proteins.^235^^,^^236^^,^^237^^,^^238^ In CVDs, specific lncRNAs have emerged as crucial players.^239^^,^^240^^,^^241^^,^^242^ One example is the antisense non-coding RNA in the INK4 locus (ANRIL), residing at the 9p21 locus, which serves as a scaffold to regulate cyclin-dependent kinase inhibitor 2a/B expression, perpetuating atherosclerosis.^243^^,^^244^ Another lncRNA, NEAT1 is involved in atherosclerosis progress by inducing lipid accumulation and inflammation responses via miR-342-3p.^245^ LncRNA RP11–728F11.4 plays a pro-atherosclerotic role by increasing the expression of FXYD6, resulting in intracellular cholesterol buildup and elevated production of proinflammatory cytokines.^246^ On the contrary, CARMN functions as an anti-atherogenic lncRNA by recruiting the PRC2 complex in cardiomyocytes. It transactivates the myocardin/SRF master regulator of vascular smooth muscle cell differentiation, thereby preserving the contractile smooth muscle cell state and offering protection against atherosclerotic neointima growth in blood vessels.^247^

In addition to their impact on atherogenicity, lncRNAs are involved in other aspects of CVDs as well. For instance, the lncRNA cardiac physiological hypertrophy-associated regulator (CPhar) is associated with exercise-induced cardiac hypertrophy. CPhar overexpression in mice induces cardiac hypertrophy and proliferation, and reduces apoptosis during ischemia-reperfusion injury by sequestering CCAAT/enhancer-binding protein β and regulating targets like ATF7.^248^ Other notable examples include (myocardial infarction-associated transcript) and H19 variants, which are linked to myocardial infarction and increased CAD, respectively.^249^^,^^250^ LncRNAs such as CHROME, MALAT1 (metastasis-associated lung adenocarcinoma transcript 1), and H19 also contribute to lipid metabolism by regulating SREBP and LXR transcription factors.^251^^,^^252^^,^^253^ Additionally, lncRNAs, like APOA1-AS and APOA4-AS, influence lipoprotein metabolism through positional expression within the human apo gene cluster.^254^ In terms of vasculature biology, MALAT1 regulates angiogenesis, while H19 induces aneurysm formation and regulates endothelial cell aging.^255^^,^^256^^,^^257^ Other lncRNAs like PUNISHER, MEG3, and GATA6-AS contribute to angiogenesis, while lncRNA-p21 restricts smooth muscle cell proliferation and is also downregulated in a mice model of atherosclerosis.^258^ In cardiac hypertrophy and heart failure, Mhrt is reported to be protective against pathological hypertrophy, while Chast and Chaer are upregulated.^259^^,^^260^ These examples highlight the intricate involvement of lncRNAs in diverse biological processes underlying CVDs, offering potential avenues for therapeutic interventions. Furthermore, the high tissue specificity of lncRNAs, ranging from 51% to 63%, distinguishes them from mRNAs (19% specificity), suggesting that drugs targeting tissue-specific lncRNAs may reduce off-target effects and enhance therapeutic precision.^261^^,^^262^

### Current clinically tested or approved RNA therapeutics for CVD

Current RNA therapeutics for CVD are primarily focused on reducing circulatory lipoprotein levels, as hypercholesterolemia poses a significant risk for CVD. Early RNA therapies like Mipomersen, a second-generation phosphorothioate ASO targeting apolipoprotein B-100, were approved for homozygous FH but discontinued due to severe liver toxicity.^263^^,^^264^^,^^265^ Newer generations of ASOs, such as Volanesorsen and Pelacarsen, have been developed with reduced toxicity through chemical modifications. Volanesorsen, targeting apolipoprotein C-III, shows efficacy in reducing TG levels by over 70% in patients with familial chylomicronemia syndrome and hypertriglyceridemia during phase III trials, but it comes with a risk of thrombocytopenia.^266^^,^^267^ To address this issue, Olezarsen and ARO-APOC3, GalNAc-bound RNAi agents, are novel agents in development.^268^ In a phase I study, ARO-APOC3 demonstrated safety and consistent reductions in APOC3, TG, and non-HDL-C, regardless of the underlying genetic cause of severe hypertriglyceridemia.^269^ Currently, phase II and III trials are ongoing for both agents in patients with hypertriglyceridemia and familial chylomicronemia syndrome.

Pelacarsen (also known as TQJ230), an siRNA molecule, targets lipoprotein(a) (LPA), a known risk factor for CVD and aortic stenosis.^270^ In a phase II trial, pelacarsen demonstrated significant dose-dependent reductions in LPA levels in patients with CVD.^271^ Alternatively, olpasiran, another siRNA molecule, has been used to disrupt LPA expression, showing dose-dependent reductions in LPA levels.^272^^,^^273^ Additionally, RNA-based therapeutics targeting *PCSK9* and angiopoietin-like 3 (*ANGPTL3*) have shown promising results. Inclisiran, a GalNAc-modified siRNA targeting *PCSK9*, has consistently shown both effectiveness and a positive safety profile, leading to its approval for treating FH and atherosclerotic CVD.^274^^,^^275^^,^^276^^,^^277^ Another siRNA, ARO-ANG3, targeting *ANGPTL3*, an inhibitor of lipoprotein metabolism, has successfully reproduced the genetic effects of familial combined hypolipidemia by lowering ANGPTL3, TG, VLDL-C, and LDL-C in a phase I trial.^278^ Aside from addressing circulatory lipoprotein, a locked nucleic acid-based ASO inhibitor of miR-132 (anti-miR132; CDR132L) has demonstrated preclinical efficacy and safety in chronic heart failure following myocardial infarction. It has since completed evaluation in a phase 1b study (NCT04045405) and advancing to a phase 2 trial (NCT05350969).^63^

Apart from ASOs and siRNA, mRNA therapeutics have emerged as a promising approach for CVD. Notable candidates include mRNA-0184, encoding a relaxin fusion protein, and AZD8601, encoding VEGF-A.^279^ The relaxin fusion protein encoded by mRNA-0184 plays a vital role in cardiovascular remodeling, offering protection against vascular strain and promoting cell growth and survival, while demonstrating improved pharmacology with the potential to increase protein expression and prolong half-life compared with its predecessor, serelaxin.^280^ To address myocardial ischemia, AZD8601 encodes VEGF-A to promote cardiac function recovery by stimulating partial tissue regeneration and enhancing blood vessel growth.^281^

Furthermore, the shift toward achieving permanent genomic editing is gaining momentum with the evolving clinical CRISPR landscape. A significant development in this direction is the phase 1B trial initiated by Verve Therapeutics in 2022, which focuses on addressing heterozygous FH. This trial employs a base editor known as VERVE-101, delivered via LNPs, to orchestrate an A to G nucleotide alteration in the PCSK9 gene to permanently silence its expression, thereby mitigating the impact of disease-propelling LDL-C levels.^282^ The liver, a primary source of the target protein, is chosen for its innate liver affinity, aligning with the suitability of LNP delivery. Importantly, usage of LNPs also ensures the transient presence of CRISPR components, minimizing the risk of unintended changes.

The various stages of clinical trials for different RNA therapeutics, as shown in Table 2, demonstrate the significant progress in harnessing RNA-based treatments to revolutionize CVD management. These advancements hold tremendous promise in transforming CVD treatment and providing exciting prospects for enhanced cardiac care.

**Table 2:** Clinically tested RNA therapeutics

Clinically tested RNA therapeutics										
Name	Target	Condition	Phase 1		Phase 2		Phase 3	Completed		NCT number
**Antisense Oligonucleotides**										

Volanesorsen	Apolipoprotein C-III	Hypertriglyceridemia Familial Chylomicronemia Syndrome Lipoprotein Lipase Deficiency Hyperlipoproteinemia Type 1	✓	✓		✓			NCT02658175, NCT02300233, NCT02211209, NCT02910635, NCT05185843, NCT01529424	
Olezarsen	Apolipoprotein C-III	Hypertriglyceridemia Severe Hypertriglyceridemia Familial Chylomicronemia Syndrome Atherosclerotic Cardiovascular Disease	✓	✓		✓			NCT05681351, NCT05130450, NCT05185843, NCT05610280, NCT05552326, NCT05079919, NCT05355402, NCT04568434	
Pelacarsen	Lipoprotein (a)	Cardiovascular Diseases Acute Coronary Syndrome Aortic Stenosis Hyperlipoproteinemia	✓	✓		✓			NCT05900141, NCT04993664, NCT05646381, NCT05305664, NCT03070782, NCT04023552	
CDR132L	miR132	Heart Failure	✓	✓					NCT04045405, NCT05350969, NCT05953831	

**SiRNA**										

Olpasiran	Lipoprotein (a)	Atherosclerotic Cardiovascular Disease Cardiovascular Disease Elevated Serum Lipoprotein(a)	✓	✓		✓			NCT04987320, NCT05581303, NCT04270760	
ARO-APOC3	Apolipoprotein C-III	Dyslipidemia Mixed Dyslipidemia Hypertriglyceridemia Severe Hypertriglyceridemia Familial Chylomicronemia Syndrome	✓	✓		✓			NCT05413135, NCT05089084, NCT04998201, NCT04720534, NCT03783377	
ARO-ANG3	Angiopoietin-like protein 3	Dyslipidemia Mixed Dyslipidemia Hypertriglyceridemia Homozygous Familial Hypercholesterolemia	✓	✓					NCT04832971, NCT05217667, NCT03747224	
Inclisiran	PCSK9	Atherosclerotic Cardiovascular Disease Coronary Artery Disease Heterozygous or Homozygous Familial Hypercholesterolemia Primary Hypercholesterolemia Mixed Dyslipidemia Acute Coronary Syndrome	✓	✓		✓		✓	NCT04929249, NCT05682378, NCT05362903, NCT05118230, NCT05726838, NCT05399992, NCT04873934, NCT05888103, NCT05639218, NCT04652726, NCT04659863, NCT03814187, NCT03060577, NCT03705234, NCT03397121, NCT05004675, NCT03400800, NCT03399370, NCT04807400, NCT05192941, NCT02597127, NCT04774003, NCT03851705, NCT04666298, NCT04765657, NCT05763875, NCT02963311, NCT05834673, NCT05030428, NCT05360446, NCT05974345, NCT02314442, NCT05739383, NCT05438069, NCT04073797, NCT05587621, NCT05870657	

**mRNA Therapeutics**										

AZD8601	Vascular Endothelial Growth Factor (VEGF-a)	Heart Failure	✓	✓					NCT03370887	
mRNA-0184	Relaxin	Chronic Heart Failure	✓	✓					NCT05659264	

**CRISPR**										

VERVE-101	PCSK9	Heterozygous Familial Hypercholesterolemia Atherosclerotic Cardiovascular Disease	✓						NCT05398029	

## Conclusion

In this review, the background and relevant advances to the current state of RNA therapeutics in the context of CVD are described. The field of RNA therapeutics is rapidly advancing due to the diverse nature of easily modifiable RNA effectors able to modulate gene sequences and their transcript expression, as well as their translated protein counterparts. The robust nature of RNA therapeutics gives it an edge over conventional pharmaceutics, of which a majority directly affects protein function through interaction and binding. However, RNA therapeutics does face challenges, notably the limited half-life of RNA moieties. To counter this, substantial progress has been made in RNA modifications and delivery vectors. Another critical consideration is the precise delivery of RNA effectors to specific cellular compartments within the appropriate cell type. Due to the inherent characteristics of nucleotide duplexes formed by Watson-Crick base pairing, RNA effectors exhibit specificity for intracellular targets. Yet, effectively introducing RNA therapeutics into the desired cell type, especially non-liver cells like those in the heart, remains a significant obstacle. Compounding this challenge is the issue of suboptimal intracellular localization. Overcoming these hurdles necessitates refining vector development and delivery techniques to enhance cell-specific uptake and facilitate EE. Given the multitude of therapeutics either approved or progressing through clinical trials, the prospects for this field appear exceptionally promising in the years ahead.
